# Evaluation of *Chlamydia trachomatis* Genotypes in Endocervical Specimens by Sequence Analysis of ompA Gene among Women in Tehran

**DOI:** 10.1155/2023/8845565

**Published:** 2023-07-31

**Authors:** Mohammadreza Rajabpour, Amir Darb Emamie, Mohammad Reza Pourmand

**Affiliations:** Department of Pathobiology, School of Public Health, Tehran University of Medical Sciences, Tehran, Iran

## Abstract

Tehran's actual prevalence of *Chlamydia trachomatis* (CT) and its genotypes are still unclear. Molecular typing of CT strains can provide essential epidemiological knowledge and contribute to improved control measures. In this study, we aimed to determine the prevalence of CT and its genotypes in the endocervical infections of females who attended the gynecology and infertility clinics in Tehran. A total of 291 women were tested for chlamydial infection by in-house PCR using specific primers for the CT cryptic plasmid. Nested PCR for amplification of the ompA gene in positive samples was carried out, genotyping was performed by sequencing this gene, and further phylogenetic analysis was conducted. Sexual infection by CT was observed in 10.3% (30/291) of the subjects, and the mean age of patients was 30.4. The ompA gene was sequenced in 27 samples, revealing E genotypes 40.7%, (*n* = 11), F 25.9%, (*n* = 7), G 18.5%, (*n* = 5), D 11.1%, (*n* = 3), and K 3.7%, (*n* = 1). This study emphasizes the importance of the diversity among CT genotypes in our studied population and the need for wide-screening the neglected bacterial infection among women in Tehran.

## 1. Introduction

*Chlamydia trachomatis* (CT) is the most widespread bacterial sexually transmitted infection (STI) worldwide, with nearly 131 million diagnosed cases annually. It has been estimated that 4.2% of females worldwide are infected, and approximately 80% of them are asymptomatic, so it remains undiagnosed [1]. This undetected disease can make it very difficult and sometimes impossible to treat the infection properly, and severe symptoms and sequelae for females may occur, which include cervicitis, salpingitis, and Pelvic Inflammatory Disease (PID). This infection is also the most common cause of preventable infertility in women and is a risk factor for other STIs [2].

The major outer membrane protein (MOMP) of CT is one of the cell wall components, and it plays a crucial role in the infectious elementary body and intracellular reticulate body [3]. The MOMP is encoded by the ompA gene, which exhibits numerous variations in its DNA sequence [4]. These variations are localized mainly in four regions, called variable domains (VD 1–4). Three of the VDs are surface exposed and contain antigenic peptides. Serotyping using antibodies is specialized for these VDs and differentiates 15 serovars [5]. Sequencing of the ompA gene is the method of choice for genovar determination and can discriminate between 19 genotypes [6]. These genotypes and related variants (A, B/Ba, C, D/Da, E, F, G/Ga, H, I/Ia, J, K, L1, L2, L2a, and L3) can cause various types of diseases depending on serovars involved. The A, B, Ba, and C genotypes cause trachoma, while the D, Da, E, F, G, Ga, H, I, Ia, J, and K are associated with urogenital infections, and the L1, L2, L2a, and L3 genotypes cause lymphogranuloma venereum [7]. Moreover, various mutations led to the emergence of different genotype variants worldwide [4, 8, 9].

In Iran, the true prevalence of CT and the genotypes present in different regions are still unclear, making it difficult to understand the burden of the disease and preventing the creation of effective CT screening programs [10]. Molecular typing can provide necessary epidemiological knowledge and help to improve control measures [11].

Here, we aimed to determine the frequency of CT and its genotypes in the endocervical infections of females who attended the women's hospital in Tehran and evaluate the correlation between genotypes and demographic information and clinical manifestations.

## 2. Materials and Methods

### 2.1. Sample Collection

In this study, 291 endocervical samples were obtained from women who attended the gynecology and infertility clinics of two women's hospitals in Tehran from December 2018 to July 2019. Written informed consent was obtained, and a questionnaire was filled out for each patient. For specimen collection, sterile Dacron swabs were used and taken into PBS, which were transferred to the laboratory within 1 hour.

### 2.2. Detection of CT

DNA extraction was performed using Favorprep™ tissue genomic DNA extraction mini kit (Favorgen Biotech Corporation, Taiwan) according to the manufacturer's protocol. PCR for CT detection targeting a 200 bp fragment in the cryptic plasmid was performed as previously described with a few modifications [12] (Table 1). Amplification was performed in a final volume of 25 *μ*l containing a 0.5 *μ*l of each primer, 12.5 *μ*l of Taq DNA Polymerase Master Mix (Ampliqon), and 1 *μ*l of the sample DNA. The amplification program consisted of the first cycle of a 6 min denaturation at 95°C, followed by 35 cycles, each lasting 30 s at 94°C, 30 s at 55°C, and 30 s at 72°C, with a final extension of 10 min at 72°C. One sample of DNA which was confirmed by sequencing to be the CT DNA was used as a positive control in the process. It is also worth mentioning that PCR for a chromosomal target (ompA) was also carried out to make sure there is no selection bias in the positive samples.

### 2.3. Genotyping of CT

Genotyping was performed by amplifying a 990 bp fragment of the ompA gene according to a nested PCR method that was previously described [4]. The ompA fragments obtained were purified by the FavorPrep™ GEL/PCR Purification Kit (Favorgen Biotech Corporation, Taiwan). In the next step, the products were sent to another laboratory and they were bidirectionally sequenced by the Sanger sequencing method.

In the next step, a BLAST similarity search and a phylogenetic tree analysis were carried out to comprehend the evolutionary relations between clinical strains and reference strains. Each sequence was aligned with an analogous sequence from reference strains. The strains were derived from the GenBank database: GenBank accession numbers: M58938, AF063208, M17343, X62918, X62920, X52557, X52080, AF063199, X16007, AF063200, AF063201, AF063202, AF063203, AF063204, M14738, M36533, and X55700). *Chlamydia muridarum* MoPn (M64171 was used to form an outgroup [4]. The phylogenetic tree was illustrated using the maximum-likelihood method in the MEGAX software.

### 2.4. Data Analysis

Statistical analysis was performed using SPSS version 24. The association of CT genotypes with demographic characteristics was evaluated using Pearson chi-square or Fisher exact tests. *p* < 0.05 was established as statistically significant.

## 3. Results

### 3.1. Studied Population and CT Prevalence

A total of 291 females were included in this study. They attended a university-affiliated women's hospital in Tehran. The mean age of the participants was 30.4, ranging from 16 to 40. Of the 291, 30 samples (10.3%) were positive for CT by in-house PCR. The positive control for the process was a CT clinical strain confirmed by sequencing. Among the patients with chlamydial infection, 13 (43.3%) females were in the age group 27–35 years (Table 2). However, there was no significant association between CT positivity and age.

### 3.2. Genotyping of CT Positive Samples

All 30 positive samples were subjected for nested PCR of the ompA gene, and among them, 27 were successfully amplified. These 27 ompA fragments were sequenced for genotyping.

**Figure 1:**
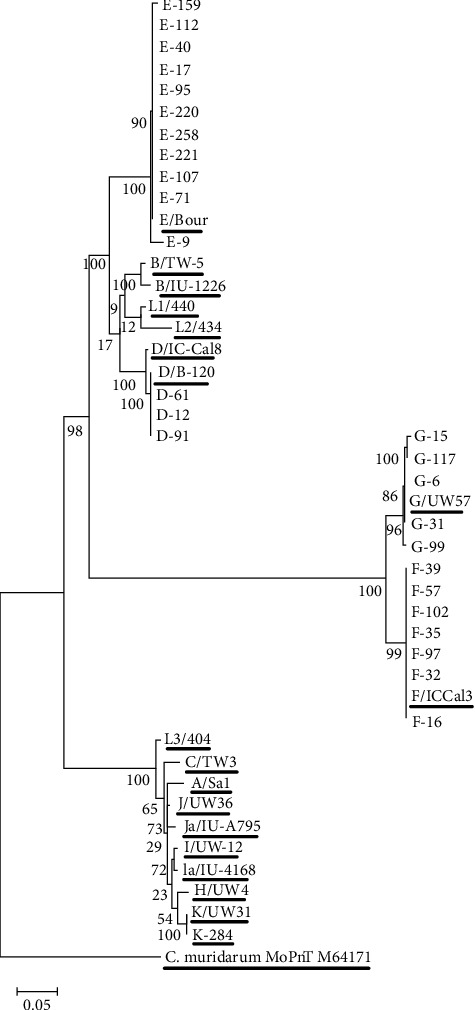
Phylogenetic tree for ompA gene nucleotide sequences. The reference sequences were underlined.

Sequence analysis revealed that the predominant genotypes were E (40.7%, *n* = 11), followed by F (25.9%, *n* = 7), G (18.5%, *n* = 5), D (11.1%, *n* = 3), and K (3.7%, *n* = 1) (Figure 1). Bootstrap values ranging from 86% to 100% confirmed the identification of the five different genotypes of CT in the samples. The ompA sequences in our study showed high similarity to the reference sequences. 5 cases of genotype E, 2 cases in genotype F, and one case in both genotypes G and D had one nucleotide change compared to reference strains. Moreover, seven nucleotide substitutions resulted in amino acid replacement (Table 3).

Patient demographic information, symptoms, and sequelae (such as vaginal discharge, infertility, and ectopic pregnancy) were analyzed for possible associations with each CT genotype, but no statistically significant associations were found (Table 4).

## 4. Discussion

In this study, the prevalence of CT infection and its genotypes among women referred to women's hospitals in Tehran were examined. To our knowledge, this is the first study investigating CT's circulating genotypes in Tehran.

CT is known as the most common bacterial sexually transmitted disease, and this fact emphasizes the importance of this investigation [1]. In this study, out of 291 patients examined, 30 were positive for chlamydial infection, 10.3% of the cases. High prevalence of this STI was reported worldwide, especially in developing countries. Huneeus et al. reported that 8.8% of the Chilean females in their studied population were infected with CT [13]. A recent study in Tanzania showed that 11% of young students (aged 18–24) were infected with chlamydia [14]. On the other hand, in Germany, only 2.8% of the females were infected with CT which shows that the frequency rates are significantly lower in developed countries [15]. According to a comprehensive study conducted in Tehran, in 2006, among 1052 women who referred to women's clinics, 12.3% were positive in terms of the presence of CT [16]. The prevalence rate in this study is not much different from the present study. In other parts of the country, different rates of chlamydial infections have been reported, which often indicates the high frequency of this bacterium. Taheri et al.'s study showed that 620 endocervical swabs were obtained from Ahvaz medical centers in October 2007 to July 2008, of which 108 positive samples (18.1%) were obtained [12]. In another study by the same authors, in 2008, endocervical samples were collected from 80 women referred to the outpatient clinic in Isfahan. The results of this study were significantly high, so 21.2% of patients were positive for CT [17]. However, the prevalence of this bacterium is not that high in all studies conducted in Iran, and some studies have reported much lower and closer numbers to the global average. In 2015, in a study by Afrasiabi et al., in Kashan, 255 married women were examined for chlamydial infection, and the microorganism was found in 2.4% of patients [18]. Comparing these numbers with each other and with the present study's findings (10.3%) shows that the study population and the differences in regions of investigation have a significant role in CT prevalence reports. Ahmadi et al., in a review study in 2015, by reviewing articles on the prevalence of CT in Iran, estimated that the prevalence of this bacterium among women in our country is 12.3% [10]. This percentage indicates the high prevalence of this infection in Iran.

Typing of CT is vital for a better understanding of the epidemiology of infection. In this study, 27 positive cases were classified into five types by analyzing the sequence of the ompA gene. Type E was found in 11 samples (40.7%), F in 7 samples (25.9%), G in 5 samples (18.5%), D in 3 samples (11.1%), and K in 1 sample (3.7%). Also, no significant relationship was found between these types and clinical signs and records. In most studies, E and F were the most common types. Studies in the Netherlands, the United Kingdom, and Australia also showed that these two genotypes are more common [19–21]. However, the prevalence of these two genotypes does not apply to all studies. For example, in a recent study by Rawre et al. In India, genotype D was the most common type among the samples; E and F were ranked next [22]. However, in another study of the same group performed on patients with infertility, genotype E had the highest rate [23]. In a study by Chen et al., in China, type D had a higher frequency than other types [24]. In the study by Brasiliense et al., conducted in 2016 in Brazil, genotype E was the most abundant, and genotypes J and F were next [25]. In the study of Köksal et al., conducted in Turkey, genotype E was the most common type, but the prevalence of type F was much lower than that of other types, corresponding to only 1.8% of cases. In this study, types G and H were common after type E in terms of prevalence [26]. These results showed that the distribution of genotypes in different geographical areas could be different, but the important point is that type E in all studies is one of the most common genotypes of this bacterium, and globally, it seems to be more common than other genotypes of bacteria.

In Iran, limited studies have been performed to determine the genotypes of CT. It is noteworthy that no study has yet examined the genotypes of this bacterium in Tehran, and the present study is the first one in this field. Taheri et al., in 2010, in Ahvaz, performed typing using the PCR-RFLP method, and genotype E was reported to be the most common bacterial type with 31.5% [12]. The same group conducted a similar study among women in the city of Isfahan, in which the genotypes E and F shared the highest frequency, and the D/Da genotype was ranked next [12]. The reason for the D/Da genotype report in this study is using the PCR-RFLP method, which does not have the power to differentiate the variants, while the sequencing method used in the present study can also identify these variants. In 2013, Saeedzadeh et al. performed the nested PCR for the ompA gene and then sequenced the amplified fragment. This study identified genotype F as the most common type, and E was next [27]. A recent study in Iran also showed that the genotype E is the most prevalent among both males and females [28]. These studies, in agreement with the present study, showed that genotypes E and F are more common in our country than in other types.

Of all the 27 ompA genes sequenced, 18 cases (66.6%) were identical to the reference strains and only 9 cases had single nucleotide substitutions. To our knowledge, these variations have not been reported in any other studies; however, no visible sign of recombination was found in our genetic variants compared to the respective reference strains, suggesting that the variations observed in our study were mainly a consequence of the point mutations.

As we interpret the results of this study, it is necessary to point out the limitations of the work. This study specifically looked for chlamydial infection among women, while a simultaneous study in men and women could help better understand the prevalence of the bacterium and its genotypes. Also, using more discriminative typing methods can lead to a better understanding of the genetic diversity of circulating bacteria. We need to mention that only single-nucleotide variations were observed in our study, and it is hard to exclude the possibility of sequencing artifacts in some cases.

This study emphasizes the importance of CT infection in the female population in Tehran. It also shows the diversity of CT genotypes in our studied population. Our results reinforce the importance of screening this neglected infection among women in Tehran.

## Figures and Tables

**Table 1:** Primer sequences used for *Chlamydia trachomatis* plasmid PCR and ompA gene PCR.

Target	Primer name	Primer sequence (5ʹ-3ʹ)	Size	References
Cryptic plasmid	CTP1	TAGTAACTGCCACTTCATC	200	[10]
	CTP2	TTCCCCTTGTAATTCGTTGC		

ompA	P1	ACTGCGTTCTGAACTGGGTG	1020	[4]
	OMP2	ACTGTAACTGCGTATTTGTCTG		

ompA (inner)	MOMP87	TGAACCAAGCCTTATGATCGACGGA	990	[4]
	RVS1059	GCAATACCGCAAGATTTTCTAGATTTCATC		

**Table 2:** *Chlamydia trachomatis* positivity rate in each age group.

Age group	CT positive (%)	Total	*p* value
16–27	11 (36.7)	75	0.38
27–35	13 (43.3)	145	
35–40	6 (20)	71	

**Table 3:** Nucleotide changes found in ompA gene of *Chlamydia trachomatis* strains which were different compared to reference sequences.

Sample code	Genotype	Nucleotide change	Position	Amino acid change
40	E	G to T	954	Silent
71	E	A to G	729	Silent
159	E	C to G	932	Thr to arg
220	E	G to A	980	Gly to asp
221	E	C to A	201	Asp to glu
35	F	C to A	878	Phe to leu
97	F	G to C	447	Ala to pro
15	G	T to G	950	Lle to ser
61	D	A to G	621	Lys to glu

**Table 4:** Prevalence of *Chlamydia trachomatis* genotypes by demographic and clinical information.

Variable	Genotype	*E* (%)	*F* (%)	*G* (%)	*D* (%)	*K* (%)	*p* value
Age group: 16–27		5 (45.4)	3 (42.8)	0 (0)	2 (66.7)	0 (0)	0.34
Age group: 27–35		3 (27.3)	2 (28.6)	4 (80)	1 (33.3)	1 (100)	
Age group: 35–40		3 (27.3)	2 (28.6)	1 (20)	0 (0)	0 (0)	

Vaginal discharge		8 (72.7)	2 (28.6)	1 (20)	2 (66.7)	0 (0)	0.15

Vulvar itching		1 (9.1)	1 (14.3)	3 (60)	0 (0)	0 (0)	0.11

Dysuria		3 (27.2)	3 (42.8)	2 (40)	1 (33.3)	0 (0)	0.89

Bleeding		1 (9.1)	2 (28.6)	0 (0)	1 (33.3)	1 (100)	0.13

Abortion		2 (18.2)	1 (14.3)	0 (0)	0 (0)	0 (0)	0.78

Ectopic pregnancy		3 (27.2)	1 (14.3)	1 (20)	0 (0)	0 (0)	0.81

Infertility		7 (63.3)	4 (57.1)	2 (40)	2 (66.7)	1 (100)	0.91

## Data Availability

All the data related to this study are available from the corresponding author upon reasonable request.

## Abbreviations

CT: *Chlamydia trachomatis*
STI: Sexually transmitted infection
PID: Pelvic inflammatory disease
MOMP: Major outer membrane protein
VD: Variable domains.
